# Comparative Study of Ex Vivo Transmucosal Permeation of Pioglitazone Nanoparticles for the Treatment of Alzheimer’s Disease

**DOI:** 10.3390/polym10030316

**Published:** 2018-03-14

**Authors:** Marcelle Silva-Abreu, Lupe Carolina Espinoza, Lyda Halbaut, Marta Espina, María Luisa García, Ana Cristina Calpena

**Affiliations:** 1 Department of Pharmacy, Pharmaceutical Technology and Physical Chemistry, Faculty of Pharmacy and Food Sciences, University of Barcelona, 08028 Barcelona, Spain; marcellesabreu@gmail.com (M.S.-A.); lcespinoza@utpl.edu.ec (L.C.E.); halbaut@ub.edu (L.H.); m.espina@ub.edu (M.E.); rdcm@ub.edu (M.L.G.); 2 Institute of Nanoscience and Nanotechnology (IN2UB), University of Barcelona, 08028 Barcelona, Spain; 3 Departamento de Química y Ciencias Exactas, Universidad Técnica Particular de Loja, Loja 1101608, Ecuador

**Keywords:** nanoparticles, pioglitazone, PLGA-PEG, transmucosal permeations, Alzheimer’s disease

## Abstract

Pioglitazone has been reported in the literature to have a substantial role in the improvement of overall cognition in a mouse model. With this in mind, the aim of this study was to determine the most efficacious route for the administration of Pioglitazone nanoparticles (PGZ-NPs) in order to promote drug delivery to the brain for the treatment of Alzheimer’s disease. PGZ-loaded NPs were developed by the solvent displacement method. Parameters such as mean size, polydispersity index, zeta potential, encapsulation efficacy, rheological behavior, and short-term stability were evaluated. Ex vivo permeation studies were then carried out using buccal, sublingual, nasal, and intestinal mucosa. PGZ-NPs with a size around of 160 nm showed high permeability in all mucosae. However, the permeation and prediction parameters revealed that lag-time and vehicle/tissue partition coefficient of nasal mucosa were significantly lower than other studied mucosae, while the diffusion coefficient and theoretical steady-state plasma concentration of the drug were higher, providing biopharmaceutical results that reveal more favorable PGZ permeation through the nasal mucosa. The results suggest that nasal mucosa represents an attractive and non-invasive pathway for PGZ-NPs administration to the brain since the drug permeation was demonstrated to be more favorable in this tissue.

## 1. Introduction

Alzheimer’s disease (AD) is a progressive neurodegenerative disease that is considered the most common cause of dementia [1,2]. AD is characterized by a gradual decline in cognition and neuropsychiatric disorders that affect the ability to perform activities of daily living [3,4]. Chronic neuroinflammation has been described as a pathological feature which may contribute to amyloid plaque progression and neurodegeneration [5,6].

PPAR-γ is a nuclear receptor whose activation regulates genes involved in glucose homeostasis, lipid metabolism, and inflammation [7,8,9]. Recent studies have shown that PPAR-γ ligands inhibit proinflammatory gene expression, regulate amyloidogenic pathways, and exhibit neuroprotective effects [10,11,12]. Pioglitazone (PGZ) is a PPAR-γ activator that increases tissue sensitivity to insulin and is widely used to treat type 2 diabetes mellitus (T2DM) [13]. Other pharmacological effects reported for PGZ include selective suppression of the T-helper 17 (Th17) cells differentiation and improvements in overall cognition using a mouse model, suggesting that PGZ is a viable treatment option not only for T2DM but also for autoimmune diseases, inflammatory conditions, and neurodegenerative diseases such as multiple sclerosis, rosacea, and AD [14,15,16,17].

PGZ is classified as a biopharmaceutical classification system (BCS) Class II drug with low solubility and high permeability which limits its absorption rate [18]. PGZ is available in conventional tablets for oral administration [19]. However, oral delivery of this dosage form has notable disadvantages such as prolonged disintegration time, first-pass metabolism, poor solubility, and low intestinal bioavailability, consequently demonstrating the need to develop new drug delivery systems and their administration by alternative routes [20].

Drugs administered via mucosal surfaces (buccal, sublingual, nasal, and intestinal tissues) provide local and/or systemic pharmacological action [21,22]. Novel mucosal delivery systems have been developed to optimize the efficacy and safety of drugs administered by these routes. Nanostructured systems are considered the most promising strategies [23,24]. Polymeric and solid lipid nanoparticles, nanostructured lipid carriers, and nanoemulsions are examples of nanotechnologies that offer numerous benefits including improved solubility for hydrophobic drugs, controlled drug release, and enhanced stability and bioavailability [25,26]. Polymeric nanoparticles (PNPs) are extensively employed due to their favorable properties, not the least of which include their ease of manufacture, low toxicity, biocompatibility, protection of drug, and biodegradation [27,28]. PNPs are defined as particles with a size ranging from 10 nm to 1000 nm that are composed of either natural polymers (gelatin, albumin, chitosan) or synthetic polymers such as polylactides (PLA), poly(lactic-*co*-glycolic) acid (PLGA), and polyglycolides (PGA) [28,29]. The incorporation of mucoadhesive polymers that adhere to a mucosal surface prolongs the residence time at the administration site of these drug delivery systems, increasing the local or systemic bioavailability [30]. Polyethylene glycol (PEG) is a hydrophilic polymer that is non-toxic and used in many pharmaceutical formulations. Surface coating with PEG is reported to prevent non-specific interactions of serum proteins with NPs [31]. PLGA-PEG copolymer nanoparticles are composed of a hydrophilic surface of PEG around a hydrophobic core of PLGA [32]. This structure allows the encapsulation of hydrophobic drugs into the core region and prolongs the circulation time while the PEG hydrophilic shield around the particle core augments mucus-penetrating properties [33,34].

The purpose of this study was to determine the best mucosal route for the administration of NPs of PGZ on the basis of their biopharmaceutical parameters in order to provide drug delivery to the brain for optimal treatment of AD. Additionally, rheological behavior and short-term stability were analyzed.

## 2. Materials and Methods

### 2.1. Materials

PGZ was purchased from Capot Chemical (Hangzhou, China), and Diblock copolymer PLGA-PEG (Resomer^®^ Select 5050 DLG mPEG 5000–5 wt % PEG) was purchased from Evonik Corporation (Birmingham, AL, USA). Tween (Tw) 80 and acetone were obtained from Sigma-Aldrich (Madrid, Spain) and Fisher Scientific (Pittsburgh, PA, USA), respectively. The dialysis membrane MWCO 12,000–14,000 Da was obtained from Medicell International Ltd. (London, UK) and the Transcutol was obtained from Gattefossé (Barcelona, Spain). Water filtered through a Millipore MilliQ system was used for all the experiments and reagents used were of analytical grade.

### 2.2. Methods

#### 2.2.1. Preparation of NPs and Physicochemical Characterization

PGZ-loaded PLGA-PEG NPs were developed by the solvent displacement method [35]. The formulation of PGZ-NPs consists of two phases: the first is composed of the drug, dimethyl sulfoxide (DMSO), and acetone (organic phase) while the second phase consists of Tw 80 (surfactant) and water (aqueous phase). After complete solubilization of both phases, the organic phase was added drop by drop into 10 mL of the aqueous phase. Afterwards, the NPs dispersion was concentrated to 10 mL under reduced pressure (Bücchi B-480, Flawil, Switzerland).

The NPs mean size (Zav) and polydispersity index (PI) were determined by photon correlation spectroscopy (PCS) using a ZetaSizer Nano ZS (Malvern Instruments, Madrid, Spain). Measurements were carried out in triplicate at angles of 180° in 10-mm diameter cells at 25 °C. The surface charge, or Zeta potential (ZP), was calculated from electrophoretic mobility. This parameter can give information about the possibility of particles aggregation [36]. The encapsulation efficiency (EE) of PGZ in the NPs was determined indirectly following Equation (1). The non-entrapped PGZ was separated using filtration/centrifugation (1:10 dilution) with Ultracell–100 K (Amicon^®^ Ultra; Millipore Corporation, Billerica, MA, USA) centrifugal filter devices at 12,000 rpm for 15 min. PGZ was measured using a previously validated high performance liquid chromatographic (HPLC) method [15].

(1) $$EE\left(\%\right)=\frac{Total\;amount\;of\;PGZ\;-\;Free\;amount\;of\;PGZ}{Total\;amount\;of\;PGZ}\cdot 100$$

#### 2.2.2. Tissue Samples

Samples were extracted from pigs (male, weight 30–40 kg, *n* = 6) following a process supervised by veterinary officials in accordance with the Ethics Committee of Animal Experimentation at the University of Barcelona. The pigs were anesthetized with intramuscular administration of ketamine HCl (3 mg/kg), xylazine (2.5 mg/kg) and midazolam (0.17 mg/kg). Once sedated, Propofol (3 mg/kg) was administered through the auricular vein and they were subsequently intubated and maintained under anesthesia by isoflurane inhalation. In order to induce pig euthanasia, sodium pentobarbital (250 mg/kg) was administered through the auricular vein under deep anesthesia.

After the sacrifice, mucosal samples were surgically removed from buccal, sublingual, nasal, and intestinal tissues, preserved in Hank’s balanced salt solution and refrigerated until delivery to laboratory for the initiation of experiments.

#### 2.2.3. Transmucosal Ex Vivo Permeations

The study was performed in Franz diffusion cells using buccal and nasal mucosae (0.5 mm thick), sublingual mucosa (0.3 mm thick), and uncut intestinal mucosa. The tissues were used for experiments and placed between the receptor and donor compartments. An aliquot of 0.2 mL of PGZ-NPs at 1 mg/mL were placed in the donor compartment and the same volume of samples was extracted from the receptor compartment at established time intervals of 6 h and replaced with fresh receptor medium (Transcutol/water, 6:4 *v*/*v*) at 37 ± 0.5 °C under continuous stirring. The quantitative determination of permeated PGZ per unit area (μg/cm^2^) in the different tissues was analyzed six times by the HPLC method [15]. Kinetic parameters were estimated using GraphPad Prism^®^ 6.0 (GraphPad Software Inc., San Diego, CA, USA).

#### 2.2.4. Biopharmaceutical Parameters

● Determination of PGZ extracted and recovered in the tissues

After finishing the experiment, the mucosae were extracted and used to determine the amount of PGZ retained (Qr, μg PGZ/g tissue/cm^2^). The mucosae were cleaned with sodium lauryl sulphate solution (0.05%) and washed with distilled water. The permeation area was excised and weighed, then the PGZ retained was extracted with methanol (1 mL) under sonication for 20 min in an ultrasound bath. The amount of PGZ was analyzed by HPLC.

To analyze the percentage of PGZ recovered from the mucosae, 1 mL of PGZ solution (110 µg/mL) was added to the different mucosae (six replicates), and kept for 6 h at 37 ± 1 °C using a water bath. A standard solution of 1 mL PGZ at 110 µg/mL was also kept at 37 ± 1 °C for the same period as a reference.

The PGZ retained from mucosae permeation and recovery samples was quantified using a validated HPLC method [15].

● Data analysis

The cumulative amount of PGZ (µg) permeated through mucosae was plotted as a function of time (h). The slope and intercept of the linear portion of the plot was derived by regression using GraphPad Prism^®^, 5.0 version software (GraphPad Software Inc., San Diego, CA, USA).

The flux values (*J_ss_*, µg/min/cm^2^) across the mucosae and the permeability coefficients (*K_p_*) were calculated per unit surface area versus time plot. In this plot, the lag time ($T_{l}$, min) is the intercept with the *x*-axis (time), determined by linear regression analysis of the permeation data using GraphPad Prism^®^ 5.01 (GraphPad Software Inc., San Diego, CA, USA). The flux values are demonstrated by Equation (2):

(2) $$J_{ss}=\frac{Qt}{A}\cdot t$$

where *Qt* is the quantity of PGZ transferred across the mucosae into the receptor compartment (µg), *A* is the active cross-sectional area accessible for diffusion (cm^2^), and *t* is the time of exposure (min).

The permeability coefficients (*K_p_*, cm/min) were obtained by Equation (3):

(3) $$K_{p}=\frac{J_{ss}}{C_{0}}$$

where *J_ss_* is the flux calculated at the steady state and *C*_0_ is the initial drug concentration in the donor compartment.

Parameters of permeation (cm) and diffusion (min^−1^), *P*_1_ and *P*_2_, respectively, were estimated from Equations (4) and (5):

(4) $$K_{p}=P_{1}\cdot P_{2}$$

(5) $$T_{l}=\frac{1}{6}\cdot P_{2}$$

The theoretical human steady-state plasma concentration (*C_ss_*) of the drug, which predicted the potential systemic concentration achieved after mucosae administration, was obtained using Equation (6):

(6) $$C_{ss}=J_{ss}\cdot \frac{A}{Clp}$$

where *C_ss_* is the plasma steady-state concentration, *J_ss_* the flux determined in this study, *A* the hypothetical area of application, and *Clp* the plasmatic clearance. The calculations were based on a maximum area of application of 20 cm^2^ for buccal, 15 cm^2^ for sublingual, and 150 cm^2^ for nasal [37,38] mucosae, as well as a human Clp value of 2.26 L/h ± 1.22 [39] in order to ensure the local action of the formulation.

In addition, the mean transit time (MTT, day) of the drug in the mucosae was also obtained using Equation (7):

(7) $$MTT=\left[\frac{V_{1}}{P_{1}\cdot P_{2}\cdot A_{E}}\right]+\left[\frac{1}{2\cdot P_{2}}\right]$$

where *V*_1_ (mL) is the volume of the donor compartment and *A_E_* (cm^2^) is the area of experimental mucosae samples.

#### 2.2.5. Rheological Behavior

The PGZ-NPs were placed in glass vials with rubber tops and aluminum capsules, then stored at room temperature (23 ± 3 °C). Rheological properties were determined at *t*_0_ = 24 h after NPs preparation using a rotational Haake RheoStress 1 rheometer (Thermo Fischer Scientific, Karlsruhe, Germany) connected to a temperature control Thermo Haake Phoenix II + Haake C25P and equipped with cone-plate geometry (0.105 mm gap) including a Haake C60/2Ti mobile cone (60 mm in diameter and 2° angle). The temperature was adjusted to 25 °C. PGZ-NPs were tested in two replicates, each undergoing a program consisting of a Three-Step Shear Profile: firstly, a ramp-up period from 0 s^−1^ to 50 s^−1^ over a 3-min span, followed by a constant shear rate period at 50 s^−1^ for 1 min, and finally the ramp-down period from 50 s^−1^ to 0 s^−1^ for 3 min. The data from the flow curves (*τ* = ($\dot{\gamma }$)) were fitted to different mathematical models. The equations are summarized in Table 1.

Viscosity mean value at *t*_0_ and 25 °C was determined from the constant shear stretch at 50 s^−1^ of the viscosity curves (*η* = ($\dot{\gamma }$)). The determination of the disturbance of the microstructure during the test or “apparent thixotropy” (Pa/s) was also evaluated.

#### 2.2.6. Short-Term Stability

The PGZ-NPs were analyzed for their stability at 4 °C and 25 °C by light backscattering and transmission profiles using Turbiscan^®^Lab Formulaction (Toulouse, France). A glass measurement cell was filled with 20 mL of formulation. The radiation source was a pulsed near-infrared light and was received by transmission and backscattering detectors at angles of 90° and 4° from the incident beam, respectively. Data were analyzed once a month for 24 h at 1-h intervals over a period of three months.

#### 2.2.7. Statistical Analysis

The statistical analysis of the permeation studies was made using GraphPad Prism^®^ 6.0 (GraphPad Software Inc., San Diego, CA, USA). The values were expressed as averages ± SEM. The software packages Haake RheoWin^®^Job Manager V.3.3 and RheoWin^®^Data Manager V.3.3 (Thermo Electron Corporation, Karlsruhe, Germany) were used to carry out the testing and analysis of the obtained rheological data, respectively.

## 3. Results

### 3.1. Physicochemical Characterization

After previous factorial design, the PGZ-NPs showed a size around 160.0 ± 1.3 nm with PI values in the range of monodisperse systems (PI < 0.1) and high association efficiency (≈92%). Moreover, the ZP was −13.9 mV, which is indicative of the stability of these systems [41].

### 3.2. Ex Vivo Permeation Studies

**Figure 1:**
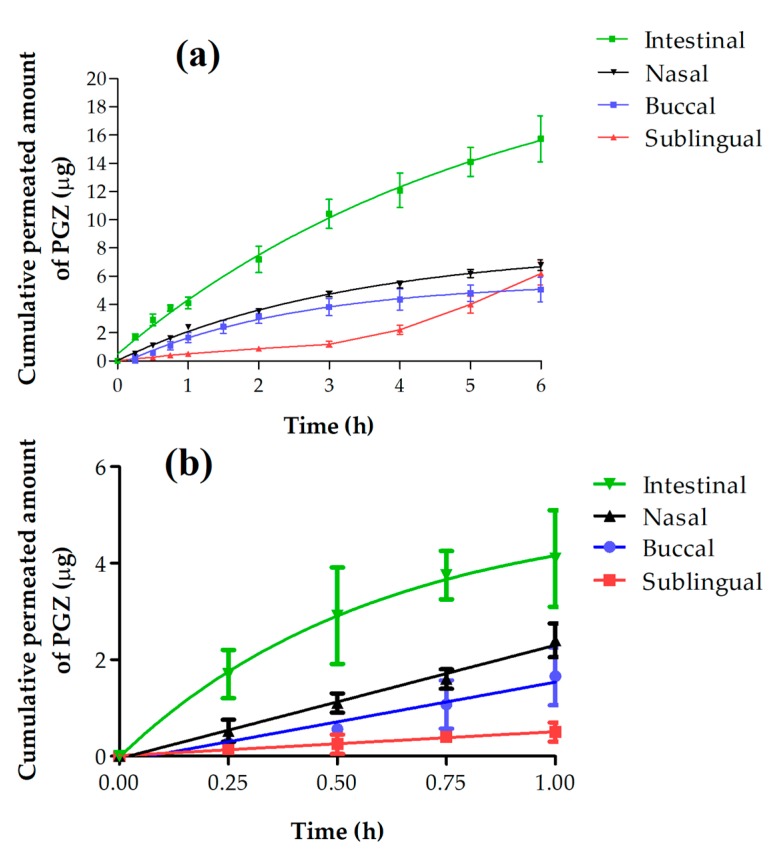
(**a**) Cumulative permeated amount of Pioglitazone (PGZ) within 6 h; (**b**) Cumulative permeated amount of PGZ within 1 h.

Figure 1a shows the permeation profile of PGZ (µg) from NPs in buccal, sublingual, nasal, and intestinal mucosae. This revealed that the cumulative permeated amount of PGZ after 6 h of assay was higher in intestinal mucosa with a value of 15.40 µg, while buccal, sublingual, and nasal mucosae presented values of 5.06, 6.20, and 6.80 µg, respectively. The permeability parameters were calculated for all mucosae studied except intestinal mucosa because it did not show a linear stretch, which is necessary to calculate these parameters (Figure 1b).

#### 3.2.1. Retained Amount of PGZ

**Figure 2:**
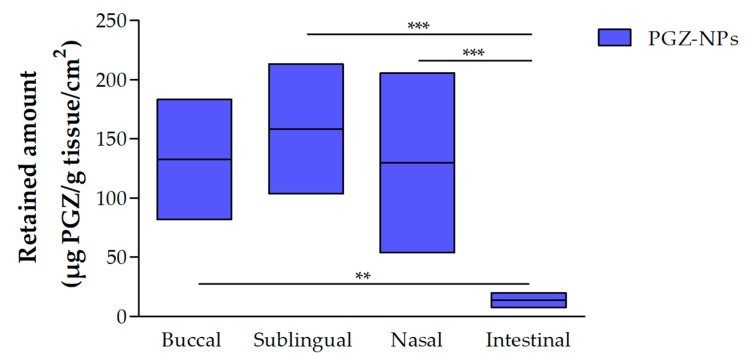
Retained amount of PGZ from nanoparticles (NPs) in different tissues. (*n* = 6). One-way analysis of variance (ANOVA) with Tukey’s multiple comparison tests were performed to assess the statistical significance (*p* < 0.05).

The NPs showed Significant Statistical Differences (SSD) (*p* < 0.05) in all tissues, except buccal between sublingual mucosa (Figure 2). The highest retained amounts were obtained by sublingual and buccal mucosa with median values of 158.45 and 132.66 (µg PGZ/g tissue/cm^2^), respectively. The nasal mucosa presented values of 129.81 (µg PGZ/g tissue/cm^2^). The intestinal mucosa showed a low retained amount of PGZ compared with the other mucosae. The percentage of recovery calculated experimentally for each tissue were: buccal 34.84%; sublingual 32.73%; nasal 37.52%; intestinal 14.87%.

#### 3.2.2. Permeation and Predictions Parameters Data

Table 2 shows the permeation and prediction parameters of PGZ from NPs through different mucosae. It was observed that *J_ss_* and *K_p_* showed similar values between all studied mucosae without SSD (*p* > 0.05). Concerning Tl, sublingual mucosa showed a value of 175.60 min, followed by buccal and nasal mucosae with values of 41.21 and 3.0 min, respectively. These results revealed an SSD between nasal mucosa with respect to buccal and sublingual mucosa, suggesting that nasal administration makes possible the achievement of state of steady equilibrium in the shortest time. With respect to the other mucosae studied, nasal mucosa also showed an SSD with the lowest values of vehicle/tissue partition coefficient (P1) and the highest values of diffusion coefficient (P2) and *C_ss_*.

The value of *C_ss_* in the nasal mucosa was 10 times greater than the other mucosae studied, signifying that PGZ administered through this route would achieved greater concentrations of PGZ in the bloodstream (relative to the other tissues).

### 3.3. Rheological Study

**Figure 3:**
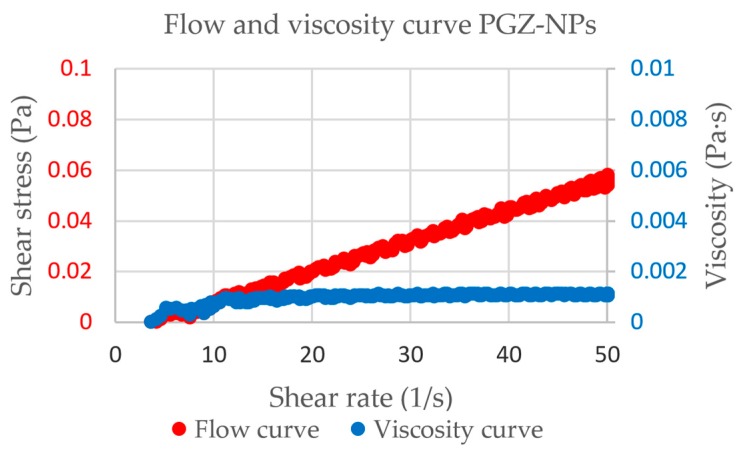
Rheograms obtained for the PGZ-NPs.

Flow and viscosity curves are depicted in Figure 3. Table 3 displays the results obtained from the rheological characterization of PGZ-NPs. The flow curves indicated no thixotropic behavior in the system since the rheograms did not exhibit hysteresis loop. The mathematical model that provided the best overall match of experimental data based on the highest correlation coefficient of regression (*r*) was the Newton model. PGZ-NPs showed a viscosity of 1.110 mPa·s.

### 3.4. Short-Term Stability

**Figure 4:**
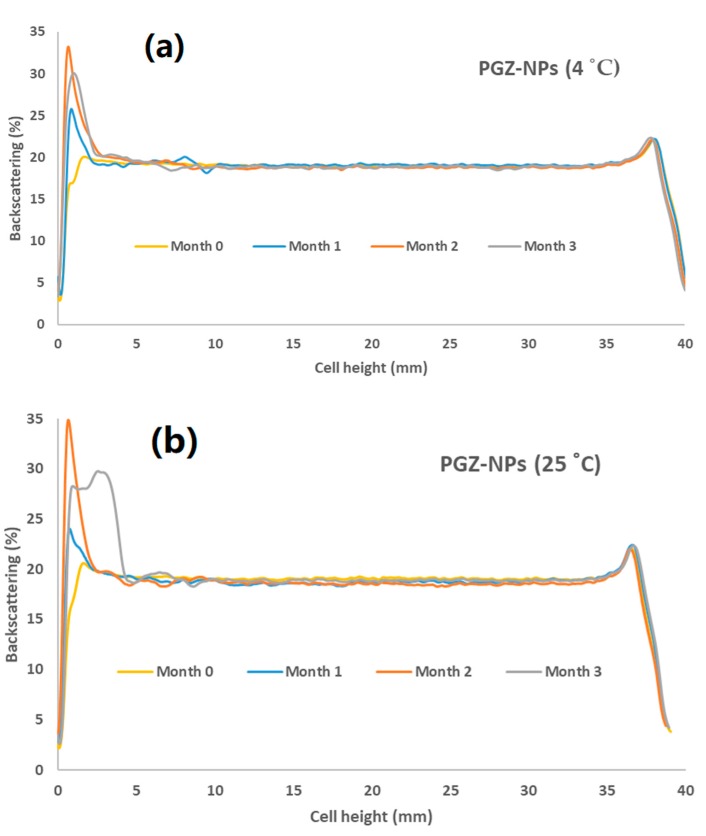
Stability of PGZ-NPs: (**a**) 4 °C and (**b**) 25 °C.

Figure 4a,b show the backscattering profiles of PGZ-NPs at 4 °C and at 25 °C for three months. In both profiles it was observed that after the first month there was an increment of sedimentation and after the second month the samples became unstable with a difference of backscattering above 10%.

## 4. Discussion

Currently, AD remains incurable and the pharmacological options have notable disadvantages such as conventional dosage forms exclusively for oral administration, which then cause discomfort among geriatric patients who have difficulty swallowing; being limited to only treat the cognitive symptoms; first-pass metabolism; and ineffective ability to cross the blood-brain barrier (BBB) [42,43]. Recently, the anti-inflammatory and neuroprotective effects of PPAR-γ ligands coupled with the advantages of nanotechnology-based drug delivery systems have come to represent a breadth of new possibilities in the treatment of AD [5,44,45]. By taking into account the fact that PGZ metabolizes in the liver and has low solubility, which limits the absorption rate, it can be concluded that it is necessary to optimize the delivery of the therapeutic product to the brain by designing a more appropriate drug delivery system and determining the most effective administration route. The PGZ-loaded PLGA-PEG NPs obtained in this study represent a promising strategy to facilitate the delivery of drugs to the brain. The physicochemical evaluation of this formulation showed favorable properties for the penetration of the drug across the blood-brain barrier (BBB) and the delivery of the drug in a controlled and sustained manner. Such advantages include small size (160.0 ± 1.3 nm), high association efficiency (≈92%), and good stability [41,46]. In addition, NPs are generally advantageous because of their good biocompatibility, capacity to adjust drug release, and remarkable enhancement of efficacy and bioavailability [29,47,48]. The surface coating of PNPs with PEG provides an increase in circulation lifetime and an improvement of drug delivery across the BBB [49]. The ability of NPs to cross biological membranes is influenced by size, shape, NP composition, and surface properties. The exact mechanism by which NPs cross lipid bilayers remains unknown because of the complexity of both NPs and cell membranes. Nanotoxicity and cell plasma membrane disruptions are concerns of NP designers [50,51]. Hypotheses such as endocytosis, the formation of nanoscale membrane holes, or membrane translocation have been proposed. Some studies support the idea that NPs cross the cell membrane via adhesive or diffusive mechanisms [51]. Clearly, further studies are required in order to assure the success of biomedical applications of these delivery systems.

The ex vivo permeation studies of PGZ-loaded PLGA-PEG NPs through different mucosae (Figure 1a) revealed that intestinal mucosa had the highest amount of drug permeated at 6 h of the assay (15.40 µg) followed by nasal (6.80 µg), sublingual (6.20 µg), and buccal (5.06 µg) mucosa. The high permeability of this formulation in all mucosae is likely due to its nano-size structure and lipophilic nature, which confers larger specific surface area and has a permeation-enhancing effect [52]. Although the amount of PGZ permeated through the intestinal mucosa was higher, it is important to consider that this route has notable disadvantages in the drug delivery to the brain, including first-pass metabolism. Gastrointestinal drug degradation constitutes one of the causes of the poor bioavailability of therapeutic agents using this route [53].

The permeation and prediction parameters (Table 2) were calculated for all of the mucosae studied except for intestinal mucosa, because the permeation profile of this mucosa did not show a linear stretch necessary for the calculation of these parameters. The values of *J_ss_* and *K_p_* obtained for buccal, sublingual, and nasal mucosa were similar without SSD (*p* > 0.05). However, Tl for nasal mucosa (3 min) was significantly lower with respect to buccal (175.60 min) and sublingual mucosae (41.21 min), which indicates a rapid onset of action using the nasal route [54]. Moreover, the estimated vehicle/tissue partition coefficient (P_1_) for nasal mucosa was lower compared with that of other mucosae, whereas the diffusion coefficient (P_2_) and *C_ss_* were higher, demonstrating that PGZ permeation is more favorable in this tissue and consequently that there is greater probability to deliver effective concentrations of PGZ at the site of action more quickly [55]. These results suggest that nasal mucosa represents an attractive and non-invasive method for drug delivery to the brain [56]. Nasal physiology and histology is characterized by high vascularization, large absorptive surface area, the avoidance of first-pass metabolism, and a porous and endothelial membrane, all of which provide important advantages to deliver drugs to the central nervous system (CNS) [57]. Furthermore, the nasal passage offers direct transport from the nasal cavity to the brain and is painless and uncomplicated for drug administration [58]. The correct formulation of the dosage form is essential for pharmacological therapy by intranasal administration with the aim of avoiding the elimination of the drug through nasal mucociliary clearance [45]. PGZ-loaded PLGA-PEG NPs as a drug delivery system provide several advantages such as rapid drug permeation, drug protection, and prolonged retention at the site of drug absorption for a suitable period of time [55,59].

The rheogram of PGZ-NPs (Figure 3) shows a linear relationship between the shear stress and the strain rate, which is characteristic of Newtonian behavior [60]. Considering the nasal mucosa as the best mucosa for drug administration, this rheology and the low viscosity obtained (about 1 mPa·s, similar to the water) are ideal for nasal spray application of the formulation [61].

For the stability assay, the PGZ-NPs showed incremental yet stable sedimentation up to the second month, after which the sample became unstable with a difference of backscattering exceeding 10% (Figure 4). This instability of particles is due to the aggregation phenomena and the limited stability of polymeric NPs in aqueous suspension is well known. These results indicate that improved long-term stability could be attained by the removal of water from the solution by lyophilization or a spray-drying technique [62,63].

## 5. Conclusions

The results obtained showed that PGZ-NPs have appropriate physicochemical characteristics to facilitate their permeability through different types of mucosa. According to the permeations and prediction parameters of these delivery systems, nasal mucosa constitutes the most convenient administration route to treat AD due to the enhanced drug permeation in this tissue, resulting in a greater likelihood of achieving effective concentrations of the drug at the site of action.

## Figures and Tables

**Table 1:** Mathematical models for regression analysis.

Flow Curve—Models: $\tau =f\left(\dot{\gamma }\right)$	
Newton	$\tau =\eta \cdot \dot{\gamma }$
Bingham	$\tau =\tau_{0}+(\eta_{0}\cdot \dot{\gamma )}$
Ostwald-de-Waele	$\tau =K\cdot {\dot{\gamma }}^{n}$
Herschel-Bulkley	$\tau =\tau_{0}+K\cdot {\dot{\gamma }}^{n}$
Casson	$\tau =\sqrt[{n}]{\left(\tau_{0}^{n}+{\left(\eta_{0}\cdot \dot{\gamma }\right)}^{n}\right)}$
Cross	$\tau =\dot{\gamma }\cdot (\eta_{\infty }+(\eta_{0}-\eta_{\infty })/(1+{\left(\dot{\gamma }/{\dot{\gamma }}_{0}\right)}^{n})$

where *τ* is the shear stress (Pa), $\dot{\gamma }$ is the shear rate (1/s), *η* is the dynamic viscosity (Pa·s), *τ*_0_ is the yield shear stress (Pa), *η*_0_ is the zero shear rate viscosity, *η_∞_* is the infinity shear rate viscosity, *n* is the flow index, and *K* is the consistency index [40]. The mathematical model was selected on the basis of the correlation coefficient value (*r*).

**Table 2:** Permeations and prediction parameters of different tissues with PGZ-NPs.

Permeation and Prediction Parameters	Buccal ^a^	Sublingual ^b^	Nasal ^c^
*J_ss_* (µg/(min/cm^2^)) × 10^2^	4.28	5.19	5.19
	(2.83–5.72)	(4.91–5.50)	(4.91–5.50)
*K_p_* (cm/min) × 10^5^	4.28	5.19	5.20
	(2.83–5.72)	(4.91–5.50)	(4.92–5.50)
Tl (min)	41.21	175.60 ^a^	3.00 ^a,b^
	(27.27–55.15)	(174.30–179.50)	(1.08–5.00)
P_1_ (cm) × 10^4^	93.74	547.37 ^a^	8.85 ^a,b^
	(93.73–93.74)	(514.35–592.73)	(3.37–16.51)
P_2_ (min^−1^)	0.004	0.0009	0.05 ^a,b^
	(0.003–0.006)	(0.0009–0.0009)	(0.03–0.15)
Mean Transit Time, MTT (day)	5.80	4.54	4.17 ^a^
	(3.84–7.77)	(4.31–4.77)	(3.95–4.41)
*C_ss_* (µg/mL)	0.02	0.02	0.20 ^a,b^
	(0.01–0.03)	(0.02–0.02)	(0.19–0.21)

^a,b,c^ Results are expressed by median, minimum, and maximum (*n* = 6). One-way analysis of variance (ANOVA) with Tukey’s multiple comparison tests were performed to assess the statistical significance between each mucosa with respect to PGZ-NPs at (*p* < 0.05).

**Table 3:** Rheological properties of PGZ-NPs.

Rheologic Parameters	PGZ-NPs
Viscosity (mPa·s) at 50 s^−1^ and 25 °C	1.110 ± 2.362 × 10^−2^
Flow behavior (best fitting model)	Newtonian Newton * (*r* = 0.9993)

*: After discarding the first and last data.
